# The computational properties of a simplified cortical column model

**DOI:** 10.1186/1471-2202-15-S1-P92

**Published:** 2014-07-21

**Authors:** Nicholas Cain, Ram Iyer, Christof Koch, Stefan Mihalas

**Affiliations:** 1 Allen Institute for Brain Science, Seattle, WA 98103, USA

## 

The mammalian neocortex generally has a repetitious, laminar structure and performs functions integral to higher cognitive processes, including sensory perception, memory, and coordinated motor output. What computations does this circuitry subserve, that might connect these unique structural elements with their biological function? Potjans and Diesmann [1] parameterize a four- layer, two cell-type (i.e excitatory and inhibitory) model of a cortical column. Beginning with their detailed model description, we implement their model using a displacement PDE (DiPDE) population statistic approach. This approach affords fast semi-analytic numerical method to solve equations describing homogeneous neuronal populations [2] (see also [3]).

**Figure 1:**
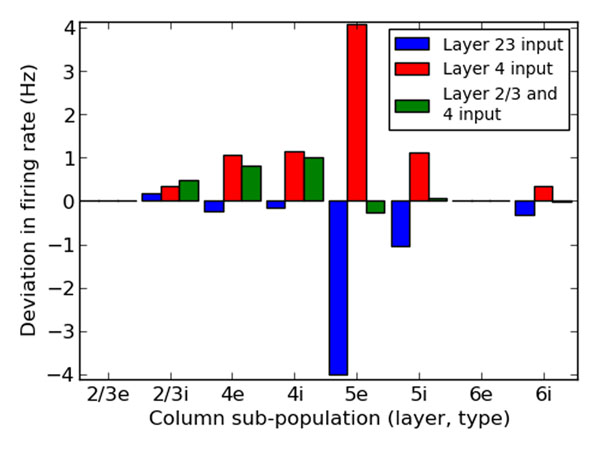
The effect of excitatory perturbations on the firing rate of subpopulations of the cortical column model. Three different excitatory perturbations of 20 Hz were applied to layers of the model. The first two excited neurons in only layer 2/3, while the third was the sum of the first two. The effect of this third perturbation was additive in the deviation of firing rate of layer 2/3 inhibitory neurons, but subtractive in layer 5 deviation.

This population statistic approach lends itself to quickly analyzing the response properties of population-scale dynamics of neural tissue. We use this strategy to examine the input-output relationship of the Potjans and Diesmann column model (see also [4]), in an attempt to uncover canonical computations [5] that it might implement. We find that excitatory perturbations to layers 4 (a site of primarily ”bottom-up” thalamic input in sensory areas) and 2/3 (a site of primarily ”top-down” cortical input) elicit an attenuated, additive perturbation in layer 23 activity, yet offset subtractively in their effect on layer 5 (see Figure 1). This computation might subserve, for example, an inferential update of prior experience with new sensory information. We generalize this finding by computing a linear kernel that describes the response of the column circuit to time varying stimuli.
